# Chemical Burn to the Eyes

**Published:** 2011-11-17

**Authors:** Justin Klaff, Stephen M Milner, Stuart Farris, Leigh Ann Price

**Affiliations:** ^a^Department of Plastic and Reconstructive Surgery, Johns Hopkins School of Medicine, Baltimore, MD; ^b^Department of Ophthalmology, Praire Eye Center, Springfield, IL

## DESCRIPTION

A 49-year-old man who sustained an alkali burn to both eyes after falling face first into a bucket of calcium hypochlorite solution (pH = 11.8) while cleaning his pool.

## QUESTIONS

1. **What is the difference in the mechanism of action between Acid and Alkali burns to the eye?**
2. **What is the immediate treatment of this injury?**
3. **How is the severity of the ocular injury determined and what are the major complications?**
4. **How are the complications managed?**

## DISCUSSION

Chemical burns to the eye represent a true emergency. Agents with the potential to cause ocular injury are often found in the home. The severity of injury correlates directly with the chemical involved, duration of contact, pH of the solution, and its penetration.^1^ Corneal epithelial damage may ultimately result in limbal ischemia and loss of limbal stem cells.^2^ The mechanism of injury and pathophysiology differs between acid and alkali burns. Alkali burn is a result of the dissociation into a hydroxyl ion and a cation in the ocular surface. The hydroxyl ion saponifies cell membrane fatty acids and causes lysis.^3^ This interaction allows deeper penetration into the corneal stroma causing denaturation of collagen and keratocyte destruction.^3^ Irreversible damage occurs at a pH value greater than 11.5. Acid burns cause protein coagulation, which prevents further penetration into the corneal stroma. Therefore, these burns are usually more superficial and do not tend to progress.^4^

Immediate, copious irrigation is the most important emergency treatment and should begin at the scene of injury^5^ (Fig 1). Morgan lenses facilitate irrigation effectively (Fig 2). A delay in irrigation is likely to result in corneal erosion and delayed healing.^5^ Sterile physiologically balanced saline solution reduces the chances of further damage to the eye; however, if this is unavailable, tap water can be substituted.^5^

The basis for assessing corneal damage is the degree of corneal opacification and perilimbal whitening^3^ (Fig 3). Although the ocular surface can recover with early management, significant sequelae include glaucoma, infection, and symblepharon. The latter defined as adhesions between the tarsal and bulbar conjunctiva. Various approaches have been used to treat these complications such as autologous conjunctival graft which can be used to restore destroyed conjunctiva.^6^ Alternatively a conjunctival flap, essentially an onlay flap of thin conjunctiva without Tenon's capsule, may be used. The exclusion of Tenon's capsule increases flap longevity. Although not favored as a primary treatment of corneal disease and trauma, they are used where lid procedures, patching, lubricants, or bandage lenses prove ineffective. Flaps placed over necrotic cornea usually become avascular, erode and are lost. Similarly, corneal perforations should be sealed prior to flap application to prevent either a continued aqueous leak or inadvertent bleb formation. Nasal mucosa has been shown to be an ideal substitute for the conjunctiva to relieve symblepharon, but its use cannot provide limbal stem cells, and recurrence is high.^6^ Amniotic membrane transplantation in acute ocular burns promotes faster healing of the epithelial defect and has greatly improved the prognosis.^7^ The amniotic membrane patch has not been shown effective in cases with severe stromal thinning and impending perforation.^7^

[To view the Surgical Procedure, Click Here]

## Figures and Tables

**Figure:**
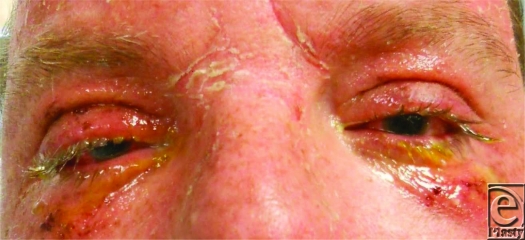


**Figure 1:**
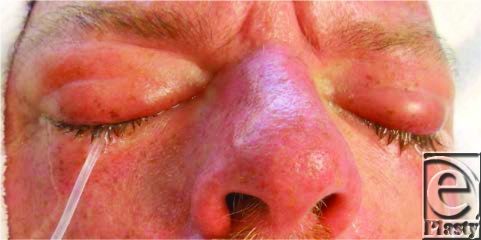
Irrigation of eye using Morgan lens.

**Figure 2:**
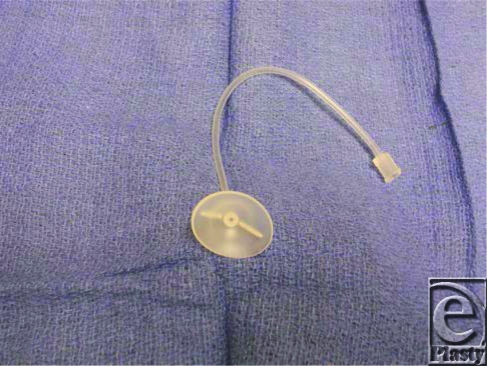
Morgan lens.

**Figure 3:**
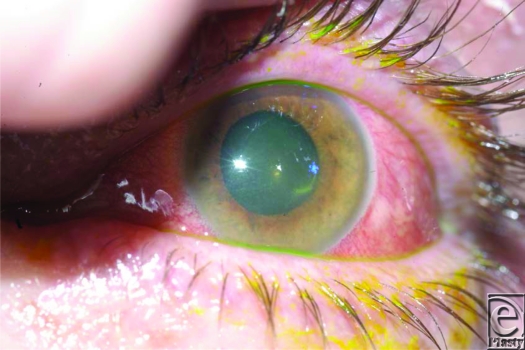
Perilimbal whitening.
